# Resistance to Ceftazidime/Avibactam in *Klebsiella pneumoniae* KPC-Producing Isolates: A Real-Life Observational Study

**DOI:** 10.3390/antibiotics12050820

**Published:** 2023-04-27

**Authors:** Laura Campogiani, Pietro Vitale, Alessandra Lodi, Alessandra Imeneo, Carla Fontana, Cartesio D’Agostini, Mirko Compagno, Luigi Coppola, Ilaria Spalliera, Vincenzo Malagnino, Elisabetta Teti, Marco Iannetta, Massimo Andreoni, Loredana Sarmati

**Affiliations:** 1 Infectious Disease Clinic, Policlinico Tor Vergata, 00133 Rome, Italy; 2 Department of System Medicine, Tor Vergata University, 00133 Rome, Italy; 3 Microbiology and BioBank, INMI Lazzaro Spallanzani, 00133 Rome, Italy; 4 Laboratory of Clinical Microbiology, Policlinico Tor Vergata, 00133 Rome, Italy; 5 Department of Experimental Medicine, University of Rome Tor Vergata, 00133 Rome, Italy

**Keywords:** *Klebsiella pneumoniae*, ceftazidime/avibactam, multidrug resistance

## Abstract

**Background:** Ceftazidime/avibactam (CAZ-AVI) resistance amongst *Enterobacterales* is worryingly increasing worldwide. **Objectives:** The aim of this study was to collect and describe real-life data on CAZ-AVI-resistant *Klebsiella pneumoniae* (KP) isolates in our University Hospital, with the ultimate goal of evaluating possible risk factors related to the acquisition of resistance. **Methods:** This is a retrospective observational study, including unique *Klebsiella pneumoniae* (KP) isolates resistant to CAZ-AVI (CAZ-AVI-R) and producing only KPC, collected from July 2019 to August 2021 at Policlinico Tor Vergata, Rome, Italy. The pathogen’s list was obtained from the microbiology laboratory; clinical charts of the corresponding patients were reviewed to collect demographic and clinical data. Subjects treated as outpatients or hospitalized for <48 h were excluded. Patients were then divided into two groups: S group, if they had a prior isolate of CAZ-AVI-susceptible KP-KPC, and R group, if the first documented isolate of KP-KPC was resistant to CAZ-AVI. **Results:** Forty-six unique isolates corresponding to 46 patients were included in the study. The majority of patients (60.9%) were hospitalized in an intensive care unit, 32.6% in internal medicine wards and 6.5% in surgical wards. A total of 15 (32.6%) isolates were collected from rectal swabs, representing a colonization. Amongst clinically relevant infections, pneumonia and urinary tract infections were the most commonly found (5/46, 10.9% each). Half of the patients received CAZ-AVI prior to isolation of the KP-KPC CAZ-AVI-R (23/46). This percentage was significantly higher in patients in the S group compared to patients in the R group (69.3% S group vs. 25% R group, *p* = 0.003). No differences between the two groups were documented in the use of renal replacement therapy or in the infection site. The clinically relevant CAZ-AVI-R KP infections (22/46, 47.8%) were all treated with a combination therapy, 65% including colistin and 55% including CAZ-AVI, with an overall clinical success of 38.1%. **Conclusions:** Prior use of CAZ-AVI was associated with the emergence of drug resistance.

## 1. Introduction

Antimicrobial resistance represents a public health problem worldwide, and carbapenem-resistant *Enterobacterales* are a serious threat, causing difficult-to-treat severe infections. In Italy, carbapenem resistance in *Enterobacterales* is endemic, mainly sustained by *Klebsiella pneumoniae* species producing different carbapenemases with KPC (*Klebsiella pneumoniae* carbapenemase) being the more common [1]. The antibiotic pipeline has focused on multidrug-resistant (MDR) pathogens in the past years, with the release of two beta-lactam beta-lactamases inhibitors in 2015: ceftazidime/avibactam and ceftolozane/tazobactam. Ceftazidime/avibactam (CAZ-AVI) is highly effective against carbapenemase-producing organisms but it is not active against class B beta-lactamases (metallo-beta-lactamases). CAZ-AVI is nowadays the first-line antibiotic to treat carbapenemase-producer *Enterobacterales*, given its documented safety and efficacy [2,3,4,5,6]. Grievously, since CAZ-AVI introduction in clinical practice in 2015, resistance to the antibiotic has been increasingly documented. The first case was reported in 2015 in a patient not previously exposed to CAZ-AVI [7], subsequent reports have incremented, leading to the release of the Rapid Risk Assessment in 2018 by the eCDC (European Center for Disease Prevention and Control) [8].

Some risk factors for CAZ-AVI resistance acquisition have been hypothesized: a retrospective study published in 2018 by Shields and colleagues identified pneumonia and the use of renal replacement therapies as major risk factors for CAZ-AVI resistance development [9], highlighting the role of sub-optimal exposure to the drug as a driver for resistance emergence [10,11]. CAZ-AVI resistance development is not yet fully understood and the identification and management of these infections still represents an important knowledge gap [12]. Aim of this study was to evaluate CAZ-AVI-resistant isolates at the Policlinico Tor Vergata University Hospital in Rome, Italy, with the ultimate goal to evaluate possible risk factors related to the acquisition of resistance.

## 2. Results

**Figure 1:**
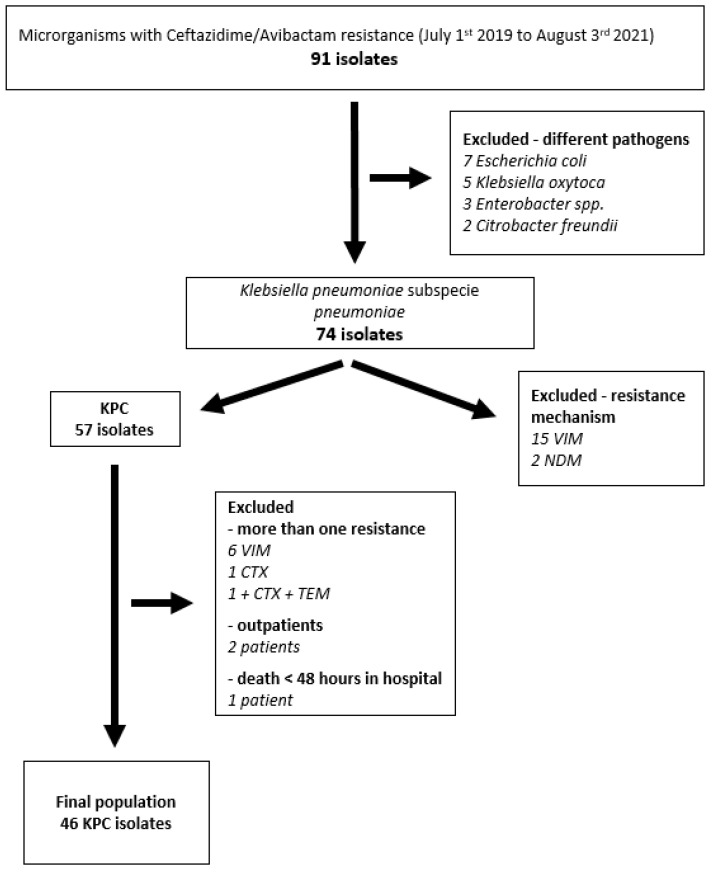
Flow chart of patients’ selection criteria.

Ninety-one CAZ-AVI-resistant (CAZ-AVI-R) *Enterobacteriaceae* isolates were collected from 1 July 2019 to 3 August 2021 at Policlinico Tor Vergata University Hospital, Rome. Seventy-four isolates (81.3%) were *Klebsiella pneumoniae*, of which seventeen (22.9%) expressed metallo-beta-lactamases, and eight expressed more than one resistance mechanism and were hence excluded. After collecting clinical data, three patients were excluded for being treated as outpatients, or hospitalized for less than 48 h, reaching a final population of 46 isolates (Figure 1).

The number of CAZ-AVI-R isolates increased over time, with only three isolates in 2019 and 31 CAZ-AVI-R strains detected until August 2021 (Table 1). Overall, 54.3% (25/46) of the enrolled patients were male, with median age of 65.5 years (interquartile range [IQR] 54.5–76). All the patients had at least one comorbidity, cardiovascular disease being the most frequent (65.2%), followed by cerebrovascular diseases (34.8%) and obesity (23.9%). Median time from hospitalization (emergency department access) to the first CAZ-AVI-R strain was 26 days (IQR 13–49.5). Most patients were hospitalized in the intensive care unit ward (28/46, 60.9%) when the CAZ-AVI-R isolate was obtained. Half of the patients received treatment with CAZ-AVI prior to the isolation of the CAZ-AVI-R KP-KPC. 

Fifteen patients (32.6%) only had rectal swab colonization, nine patients (19.6%) were colonized in different sites (six urinary tract, two skin, one respiratory system). Twenty-two patients (47.8%) had a clinically relevant infection, pneumonia and urinary tract infection being the most common (10.9% each), followed by catheter-related and secondary bloodstream infections (6.5% each) (Table 2). Thirty-two CAZ-AVI-R isolates were investigated with an immuno-chromatographic assay and 27/32 (84.4%) tested positive for the KPC enzyme. Molecular assay detected the presence of the blaKPC gene for all strains. Eleven out of forty-six (23.9%) CAZ-AVI-R isolates were susceptible to meropenem, and 4/46 (8.7%) isolates showed a meropenem minimum inhibitory concentration (MIC) in the intermediate (susceptible increased exposure) range (2 < MIC < 8 mg/L). 

Twenty patients (43.5%) had no previous *K. pneumoniae* isolate susceptible to ceftazidime/avibactam (R group), while 26 patients (56.5%) had a previous *K. pneumoniae* isolate susceptible to CAZ-AVI (S group). The two subgroups were comparable for age (median age 69.5 years old [IQR 55–77.2] R group vs. 64.5 years old [IQR 54.7–76] S group), sex distribution (males 60% R vs. 50% S), comorbidities and ward of CAZ-AVI-R strain isolation (Table 1). Median time from hospitalization to the detection of the CAZ-AVI-R isolate was significantly longer in the S group, compared to the R subpopulation (41 days [IQR 23.5–60.5] S group vs. 14 days [IQR 9–29.5] R group, *p* = 0.003). Subjects in the S group received significantly more often treatment with CAZ-AVI prior to isolation of CAZ-AVI-R strain (69.2% S group vs. 25% R group, *p* = 0.003); no differences were recorded in CAZ-AVI dosages or the use of dialysis/continuous renal replacement therapy (CRRT). As for the colonization/infection site, patients in the R group had a colonization more often (mainly rectal swab colonization), while patients in the S group had clinically relevant infections more often (*p* = 0.007) (Table 2). No differences were recorded in immunocromatography positivity rates of the CAZ-AVI-R strains. 

Data on the 22 patients with a clinically relevant infection are presented in Table 3. Two patients died within 24 h from the isolation of the CAZ-AVI-R *Klebsiella pneumoniae*, without receiving a targeted therapy. The remaining 20 patients all received combination therapy, with a median of two antibiotics. The majority of combination strategies included colistin (13/20, 65%) and ceftazidime/avibactam (11/20, 55%). Clinical success was achieved in 8/21 cases (38.1%). 

## 3. Discussion

Our study describes a cohort of 46 patients with CAZ-AVI-R *Klebsiella pneumoniae* KPC producer isolates, both as a colonization or infection, collected over a 3-year period. This is, to the best of our knowledge, the largest observational study on CAZ-AVI resistance and the first to focus specifically on *K. pneumoniae*. A recent systematic review, published in 2021 by Di Bella and colleagues, summarized available data on CAZ-AVI resistance: from 2015, a total of 24 papers were included, accounting for 42 patients with 57 strains resistant to CAZ-AVI [13]. All of them were KPC-producing, with more than 90% of *K. pneumoniae* from patients with clinically documented infections. 

Around 70% of the acquired CAZ-AVI resistance was documented in patients previously treated with CAZ-AVI, highlighting the role of antibiotic exposure as a major resistance driver. In our cohort, CAZ-AVI-R KP-KPC were isolated mainly in the Intensive Care Unit (ICU) (60.9%), probably related to a higher antibiotic use in critically ill patients, who often have an altered protein and volume distribution that could cause an altered drug exposure. Overall, half of the patients in our cohort received CAZ-AVI prior to the KP-KPC CAZ-AVI-R strain isolation, and CAZ-AVI exposure was significantly higher in the S group patients (69.2% S group vs. 25% R group, *p* = 0.003), underlining the influence of CAZ-AVI exposure in resistance development. Time from the hospital admission to CAZ-AVI-R strain isolation was significantly longer in the S group compared to the R group (41 days vs. 14 days, *p* = 0.003), again probably accounting for a greater antibiotic selective pressure as a catalyst for CAZ-AVI-R emergence. The use of CAZ-AVI in combination with other antibiotics might protect the molecule from resistance development, although so far no definitive combination therapy has been proven as more effective than others, and vivid discussion is still ongoing on the necessity of combination strategies [2,3,4,14,15]. 

CAZ-AVI resistance has also been reported in patients not previously exposed to the antibiotic [7,8,10,13] (43.5% in our cohort). The ability of the CAZ-AVI resistance genes to horizontally transfer between strains and patients might account for “baseline” CAZ-AVI resistance, calling for important national and international surveillance programs and strict infection control measures [10,16,17]. 

Some of the mutations associated with CAZ-AVI resistance, such as the D179Y, have been shown to restore meropenem activity in CAZ-AVI-resistant strains, thus phenotypically resulting as ESBLs [18,19,20]. In the present study cohort, carbapenem susceptibility has been reported in 32.6% of the CZA-AVI-R isolates (15/46; 11 [S] + 4 [I]). Data on the clinical efficacy of meropenem to treat these pathogens are still conflicting, and the risk of developing meropenem resistance during carbapenem therapy, maintaining CAZ-AVI resistance, is substantial. [13,14,18,19]. A recent literature review described a mortality of 50% and a clinical response of 62.5% in CAZ-AVI-R carbapenem-susceptible KPC-producing *K. pneumoniae* active infections treated with meropenem [14]. In our cohort, all clinically relevant CAZ-AVI-R infections were treated with a combination therapy, including carbapenemes in 40% of cases, with an overall low percentage of clinical success. New molecules such as meropenem/vaborbactam and cefiderocol might be considered [2,3,4,21,22,23], even though reduced susceptibility and cross-resistances has been reported [24,25,26]. 

Some mutations conferring CAZ-AVI-resistance have been shown to alter detectability in the available diagnostic microbiology tools, specifically rapid tests such as the immunocromatographic test, posing further challenges to both microbiologists and clinicians [14,27,28]. In the present study, only 15.6% of CAZ-AVI-R strains were not detected as KPC-producing by immunocromatographic assays (5 out of 33). Genetic characterization of the strains was performed in four isolates, and the data have been recently published [29]. The strains were all KPC-3, with D179Y substitutions in the Ω-loop; meropenem susceptibility was restored in all the isolates previously resistant to carbapenemes, and none of the strains were detected as KPC by immunocromatographic assay [29]. The identification of risk factors for the development of CAZ-AVI resistance could help select target populations in which to use specific molecular assays, genotypic testing, and phenotypic resistance profiles, changing CAZ-AVI-R *K. pneumoniae* infections management and outcome and allowing better antibiotic tailoring.

The study has several limitations, firstly due to its retrospective and monocentric design. The number of included patients is small, not allowing us to conduct robust statistical analysis. Data were collected only up to the first CAZ-AVI-R strain isolation, either as colonizer or as pathogen, so data on incidence of infections in colonized patients were not recorded, and data on antibiotic treatment and outcome of CAZ-AVI-R infections are not complete. Previous hospitalization or antibiotic treatment were not investigated, limiting observation in the R group. Genetic analysis was not performed; hence, it is not possible to infer on CAZ-AVI resistance mechanisms. However, to our knowledge, this is the largest observational study on ceftazidime/avibactam resistance in *Enterobacterales*, and the first focusing only on *Klebsiella pneumoniae* KPC in hospitalized patients, reducing possible confounding factors due to different pathogens’ mechanisms of resistance.

## 4. Conclusions

Resistance to CAZ-AVI in carbapenemase-producing *K. pneumoniae* is worryingly increasing. CAZ-AVI exposure and long hospitalization times seem to increase the risk of CAZ-AVI resistance acquisition; furthermore, CAZ-AVI-resistant KPC-producing strains are not always detected by available diagnostic tools, and susceptibility testing should be interpreted with caution. New antibiotics such as meropenem/vaborbactam might represent a valid treatment for these infections. Multicentric prospective and case-control studies are needed to better clarify risk factors for CAZ-AVI resistance acquisition during hospitalization and identify the optimal antibiotic treatment.

## 5. Materials and Methods

### 5.1. Study Design and Population Selection

The present is a retrospective cohort study, assessing prospectively collected data. Sequential unique isolates of CAZ-AVI-resistant (CAZ-AVI-R) pathogens obtained from patients admitted to the University Hospital of Tor Vergata, Rome, Italy between 1 July 2019 and the 3rd of August 2021 were included in the study. The isolate list was derived from the microbiology laboratory surveillance program for CAZ-AVI-resistant pathogens active at the study site. The study population was then restricted to *K. pneumoniae* spp. *pneumoniae* CAZ-AVI-R isolates; *Klebsiella pneumoniae* isolates in which metallo-beta-lactamases could be identified were excluded from analysis as well as bacterial isolates with more than one resistance mechanism. Finally, *Klebsiella pneumoniae* with only KPC production as their resistance mechanism were included in the study. 

The study was approved by the local ethics committee (protocol number 177.21). Given the retrospective nature of the study, patients’ informed consent was not necessary. The study was conducted in accordance with the principles of the Declaration of Helsinki.

### 5.2. Microbiology Analysis

Antimicrobial susceptibility testing was performed using ITGN Micronaut panels (Diagnostika Gmbh, Bornheim, Germany, now company of Bruker Daltonics) run on MICRO MIB (Bruker Daltonics, Billerica, MA, USA) and interpreted following the European Committee on Antimicrobial Susceptibility Testing (EUCAST) clinical breakpoint v 9.0 [30]. 

Carba SMART selective chromogenic media (bioMerieux Italia, Grassina, Italy) was used to screen for carbapenemase-producing *Enterobacterales* (CPE). Colonies detected on carba SMART from rectal swabs or on other clinical isolates were identified with MALDI-TOF MS (Bruker Daltonics). Identification of carbanemases (KPC, VIM, imipenase [IMP], NDM, oxacillin-hydrolysing [OXA-48]) was performed using the immunochromatographic (IC) assay NG CARBA (NG Biotech, Guipry, France) according to manufacturer’s instructions. Carbapenemases detected by IC assay were also confirmed by molecular assay, using an in-house-developed multiplex real time PCR probe-based assay, able to simultaneously quickly detect KPC, OXA-48, VIM and NDM [31].

### 5.3. Data Collection 

The first CAZ-AVI-R *Klebsiella pneumoniae* isolate was registered for each patient; subsequently, demographic data, comorbidities, treatment, laboratory and microbiological data were collected in an ad-hoc-created Excel database. Data were collected up until isolation of the first *Klebsiella pneumoniae* CAZ-AVI-R. Clinical data were retrieved from a clinical chart review; laboratory and microbiology findings were extracted from the electronic software of the hospital. Patients not hospitalized and those hospitalized for less than 48 h were excluded. Data on both colonization and infections were collected. The site of infection was defined according to the CDC’s National Healthcare Safety Network (NHSN) definitions, version January 2021 [32]; when NHNS criterion could not be fulfilled due to a lack of clinical information from clinical chart review, antibiotic initiation by the infectious diseases specialist to treat the CAZ-AVI-R isolate would be classified as an infection. 

The study population was divided in two subgroups: R group, if the first *Klebsiella pneumoniae* ever isolated from the patient was CAZ-AVI-R, or S group, if the patient had a prior isolation of a carbapenemase-producing *Klebsiella pneumoniae* susceptible to CAZ-AVI (with MIC below the susceptibility cut-off).

When evaluating the clinically relevant infections, clinical success was defined as the absence of signs and symptoms of infection at antibiotic interruption. 

### 5.4. Statistical Analysis

Continuous data are presented as the median with interquartile range (IQR), while categorical data are presented as frequencies with percentages. Differences between groups were assessed using the Mann–Whitney U test (two groups, continuous variable), Kruskal–Wallis test (more than two groups, continuous variable) or Chi2 test (categorical variables). Results were considered statistically significant if the *p* value was less than 0.05. Statistical analyses were performed using the software JASP (Version 0.11.1, JASP Team, 2019).

## Figures and Tables

**Table 1:** Population’s demographic characteristics, overall and by subgroups.

	Overall Population **K. pneumoniae* KPC* 46 Patients		Group R 20 Patients (43.5%)		Group S 26 Patients (56.5%)		*p*
	Absolute n°	%	Absolute n°	%	Absolute n°	%	
**Year of isolate**							
2019	3	6.5	0		3	11.5	0.269
2020	12	26.1	5	25	7	26.9	
2021	31	67.4	15	75	16	61.6	
**Age (median, IQR)**	65.5	54.5–76	69.5	55–77.2	64	54.7–76	0.816
**Sex (M/F)**	25/21	54.3/45.7	12/8	60/40	13/13	50/50	0.500
**Comorbidities**							
Cardiovascular	30	65.2	12	60	18	69.2	0.515
Cerebrovascular	16	34.8	9	45	7	26.9	0.202
Obesity	11	23.9	4	20	7	26.9	0.585
Psychiatric/dementia	11	23.9	5	25	6	23.1	0.880
Renal	11	23.9	5	25	6	23.1	0.880
Respiratory	10	21.7	4	20	6	23.1	0.802
Diabetes	8	17.4	2	10	6	23.1	0.246
Solid tumour	6	13	4	20	2	7.7	0.219
Solid organ transplant	3	6.5	0		3	11.5	0.116
Haematologic	4	8.7	2	10	2	7.7	0.783
**Days from hospital admission to CAZ-AVI-R isolate (median, IQR)**	26	13–49.5	14	9–29.5	41	23.5–60.5	**0.003**
**Ward of CAZ-AVI-R**							
Intensive care unit	28	60.9	13	65	15	57.7	0.861
Internal medicine	15	32.6	6	30	9	34.6	
Surgical wards	3	6.5	1	5	2	7.7	
**Previous CAZ-AVI treatment (yes/tot)**	23/46	50	5/20	25	18/26	69.2	**0.003**
Of which							
CAZ-AVI full dose (yes/tot)	16/23	69.5	4/5	80	12/18	66.6	0.567
CRRT/dialysis	4/23	17.4	0/5		4/18	22.2	0.246

Group R: patients with the first *K. pneumoniae* isolate resistant to CAZ-AVI; Group S: patients with *K. pneumoniae* CAZ-AVI-susceptible strains isolated prior to the CAZ-AVI-R strain. The reported *p* value was obtained using the Mann–Whitney test or the Chi2 test, as appropriate; statistically significant correlations are highlighted in bold. CAZ-AVI: ceftazidime/avibactam; CRRT: continuous renal replacement therapy; IQR: interquartile range; KPC: *Klebsiella pneumoniae* carbapenemase producer.

**Table 2:** Population’s microbiologic characteristics, overall and by subgroups.

	Overall Population *K. pneumoniae* KPC 46 Patients		Group R 20 Patients (43.5%)		Group S 26 Patients (56.5%)		*p*
	Absolute n°	%	Absolute n°	%	Absolute n°	%	
***K. pneumoniae* isolate**							
Infection	22	47.8	5	25	17	65.4	**0.007**
Colonization	24	52.2	15	75	9	34.6	
***K. pneumoniae* isolation site**							
Rectal swab colonization	15	32.6	13	65	2	7.7	
Colonization—other sites *	9	19.6	2 **	10	7	26.9	
Pneumonia	5	10.9	0		5	19.2	
Urinary tract infection	5	10.9	2	10	3	11.5	
Catheter-related BSI	3	6.5	1	5	2	7.7	
BSI (primary)	1	2.2	1	5	0		
BSI (secondary)	3	6.5	0		3	11.5	
Soft tissue and skin	2	4.3	1	5	1	3.9	
Intrabdominal infection	2	4.3	0		2	7.7	
Surgical site infection	1	2.2	0		1	3.9	
**Positive KPC** **immunocromatographic test (yes/tot)**	27/32 #	84.4	16/18	88.8	11/14	78.6	0.425
**Meropenem S**	11	23.9	5	25	6	23.1	0.737
**Meropenem I**	4	8.7	1	5	3	11.5	
**Meropenem R**	31	67.4	14	70	17	65.4	

* other colonized sites included urinary tract (6 patients), skin (2 patients), and respiratory system (1 patient). ** 2 patients with urinary tract colonization. # immunocromatographic test was performed only in 32 patients. Group R: patients with the first *K. pneumoniae* isolate resistant to CAZ-AVI; Group S: patients with *K. pneumoniae* CAZ-AVI-susceptible strains isolated prior to the CAZ-AVI-R strain. The reported *p* value was obtained with the Mann-Whitney test or the Chi2 test, as appropriate; statistically significant correlations are highlighted in bold. BSI: bloodstream infection; I: intermediate susceptibility (susceptible increased exposure); KPC: *Klebsiella pneumoniae* carbapenemase producer; R: resistant; S: susceptible.

**Table 3:** Demographic and microbiological characteristics of the patients with a KP-KPC CAZ-AVI-R clinically relevant infection.

Overall Population Clinically Relevant Infections 22 Patients		
	Absolute n°	%
**Age (median, IQR)**	54.5	57.2–74.7
**Sex (M/F)**	14/8	63.6/36.4
**Comorbidities**		
Cardiovascular	17	77.3
Cerebrovascular	5	22.7
Obesity	4	18.2
Psychiatric/dementia	4	18.2
Renal	6	27.3
Respiratory	5	22.7
Diabetes	2	9.1
Solid tumour	2	9.1
Solid organ transplant	2	9.1
Haematologic	1	4.5
**Days from hospital admission to CAZ-AVI-R isolate (median, IQR)**	28	17–49.5
**Ward of CAZ-AVI-R**		
Intensive care unit	14	63.6
Internal medicine	6	27.3
Surgical wards	2	9.1
**Antibiotic treatment**	20/22 *	90.9
Combination therapy	20	100
Number of antibiotic (median)	2	min 0–max 3
Colistin	13	65
CAZ-AVI	11	55
Carbapenem	8	40
**Days of antibiotic (median, IQR)**	10.5	4.5–14
Clinical resolution yes/no **	8/13	38.1/61.9
Death **	13	61.9

* Two patients died within 24 h from the CAZ-AVI-R isolate, no specific therapy could be started before the exitus. ** data for 21 patients, for one patient some clinical data were not retrievable. CAZ-AVI: ceftazidime/avibactam; IQR: interquartile range; KPC: *Klebsiella pneumoniae* carbapenemase producer.

## Data Availability

Data can be found in the ad hoc created Excel database, archived at the authors’ institution (Policlinico Tor Vergata Hospital, Rome, Italy).
